# Timing of reproduction modifies transgenerational effects of chronic exposure to stressors in an annual vertebrate

**DOI:** 10.1098/rspb.2022.1462

**Published:** 2022-10-12

**Authors:** Agnieszka Magierecka, Antreas Aristeidou, Maria Papaevripidou, John K. Gibson, Katherine A. Sloman, Neil B. Metcalfe

**Affiliations:** ^1^ School of Biodiversity, One Health and Veterinary Medicine, University of Glasgow, Glasgow G12 8QQ, UK; ^2^ Institute for Biomedical and Environmental Health Research, University of the West of Scotland, Lanarkshire G72 0LH, UK

**Keywords:** chronic stress, reproductive strategy, maternal allocation, offspring phenotype, transgenerational effects

## Abstract

Stressful environmental conditions can shape both an individual's phenotype and that of its offspring. However, little is known about transgenerational effects of chronic (as opposed to acute) stressors, nor whether these vary across the breeding lifespan of the parent. We exposed adult female (F0 generation) three-spined sticklebacks (*Gasterosteus aculeatus*) to chronic environmental stressors and compared their reproductive allocation with that of non-exposed controls across early, middle and late clutches produced within the single breeding season of this annual population. There was a seasonal trend (but no treatment difference) in F0 reproductive allocation, with increases in egg mass and fry size in late clutches. We then tested for transgenerational effects in the non-exposed F1 and F2 generations. Exposure of F0 females to stressors resulted in phenotypic change in their offspring and grandoffspring that were produced late in their breeding lifespan: F1 offspring produced from the late-season clutches of stressor-exposed F0 females had higher early life survival, and subsequently produced heavier eggs and F2 fry that were larger at hatching. Changed maternal allocation due to a combination of seasonal factors and environmental stressors can thus have a transgenerational effect by influencing the reproductive allocation of daughters, especially those born late in life.

## 1. Introduction

Non-genetic maternal influences have long been considered one of the major forces shaping animal phenotypes. Factors such as maternal age, size, nutritional and social status, and the choice of breeding site can have a strong influence on offspring physiology, morphology, behaviour and life-history [1–3], either as a result of adaptive maternal programming of offspring phenotype or as a side effect of the maternal phenotype, maternal environment or the combination of both [3,4]. Females living in unpredictable or stressful environments may produce offspring with phenotypic characteristics that allow them to better cope with anticipated adverse environmental conditions, although the adaptive potential of such maternal effects can be highly dependent on the matching of the maternal–offspring environments ([5,6], but see [7]). Maternal condition can be translated to offspring phenotype through a range of hormonal [8,9] and nutritional [10] factors as well as through maternally derived mRNAs [11] and epigenetic modifications [12,13]. The latter may be of particular importance in the study of inter- and transgenerational change due to the high heritability of epigenetic modifications [14]. As a result, the phenotype and/or environment experienced by mothers have the potential to affect not only their offspring but also later generations [15]. Indeed, transgenerational change due to stressful environments experienced by females encompasses more than just maternal effects [3,16] and can have major implications for the dynamics of populations and for their evolutionary potential [17,18].

A situation in which there can be far-ranging inter- and transgenerational effects arises when a female's state affects her reproductive decisions or investment. This can shape various aspects of the life-history and reproductive strategy of her offspring (the F1 generation) [19–21]. These in turn can influence the development, survival and reproductive success of her grandoffspring (the F2 generation). Thus, the effects of environmental factors (e.g. stressors) acting upon the F0 generation can be carried over to subsequent generations, even when these generations are not directly affected by the initial conditions [3].

There is increasing evidence from vertebrates that environmental factors acting upon parents may project into subsequent generations both through direct epigenetic effects [13,22] and through the alteration of the life-history and reproductive output of their offspring [21,23,24]. However, the adaptive potential of transgenerational effects spanning multiple generations remains unclear, mainly due to their high context-dependence [25]. For example, Hellmann *et al*. reported both parental and grandparental effects of predator stress, but these were sex- and lineage-specific [15,26]. Evidence of an adaptive physiological effect across generations and environmental contexts is provided by Shama *et al*., who report transgenerational plasticity to ocean warming persisting down the maternal line [27,28]. In terms of the effects on reproduction, while unfavourable environmental conditions experienced by females are often associated with reduced reproductive success of their daughters, an improvement in offspring breeding success [29] and no net effect on offspring reproduction [30,31] have also been reported. Moreover, the strength and direction of transgenerational effects may be highly dependent on the stability of the environment: if the environment experienced by daughters matches that of the mothers (i.e. is equally stressful), there may be an accumulation of phenotypic effects in later generations [3,21,24].

In contrast with effects of acute stressors, relatively little is known about the effects of chronic exposure to environmental stressors, which can be of paramount importance for the dynamics and persistence of populations in disturbed or unpredictable environments, even if the stressful conditions are only experienced by specific cohorts within a population. As opposed to acute stress, which tends to initiate an adaptive response, chronic stress can have a more deleterious effect, particularly when it is unpredictable and thus prevents habituation [32,33]; it is therefore possible that the transgenerational effects of maternal stress and their adaptive potential may be highly dependent on whether the stressful stimuli are transient or protracted.

In addition, it is not known whether the impact of environmental stressors changes over the breeding lifespan of the mother. A particular case is species that produce a sequence of clutches across a prolonged breeding season. While certain aspects of reproductive strategy show seasonal trends [34,35], these are usually attributed to changes in food abundance and temperature [34] or maternal age [36]. It is, however, unclear if and how these natural seasonal fluctuations are modified by female exposure to stressful conditions during the breeding season. McCormick [37] reported cortisol-driven, between-clutch variation in larval size at hatching using Ambon damselfish (*Pomacentrus amboinensis*), while Mileva *et al*. [38] provided evidence that eggs from successive clutches produced by stress-exposed daffodil cichlids (*Neolamprologus pulcher*) differ in size and cortisol content. However, these studies used exposure to exogenous cortisol *in ovo* and repeated exposure to the same acute stressor, respectively. Thus, it remains unclear how stressful environmental conditions experienced by F0 females interact with the timing and order of their reproductive attempts in shaping of the F1 female reproductive strategy and pre-natal development of the F2 generation. Since reproductive strategy and lifetime reproductive success of animals are crucial determinants of persistence of wild populations and of productivity in aquaculture, it is important to disentangle the effects of seasonality and environmental conditions in a context where reproduction is affected by both factors.

In this study, we explored this issue by exposing female three-spined sticklebacks (*Gasterosteus aculeatus*) from an annual population [39] to an unpredictable chronic stress protocol (UCSP) across the entire breeding season, during which they produced up to three clutches; control females also produced clutches but were not exposed to the stress protocol. The resulting offspring were reared in a non-stressful environment and allowed to breed the following year. We were therefore able to examine whether chronically stressful conditions experienced by females affected the reproductive strategy of their daughters and the developmental trajectories of their grandoffspring. The design of this study, encompassing multiple reproductive attempts of an F0 female within her single breeding season, allowed us to address the interaction between unpredictability of the environment and seasonality in shaping of the F1 and F2 phenotypes.

## 2. Methods

### (a) Fish husbandry and stress protocol

The parental F0 generation was formed from wild-caught three-spined sticklebacks from a largely annual River Endrick (Scotland) population [39], captured by netting in January 2017 and transported to aquarium facilities. In April 2017, 14 days prior to the beginning of the experiment, fish were allocated to either a UCSP-exposed (*n* = 72) or Control (non-exposed, *n* = 72) group and placed in 10 l plastic tanks: three randomly selected fish that clearly differed in size (to allow for individual identification) were placed in each of the 24 Experimental and 24 Control tanks, which were all held in the same room and received the same recirculating water supply (temperature 12°C, photoperiod 14L : 10D). For details on source population, transport and husbandry, see Magierecka *et al*. [40] and Appendix 1 of the electronic supplementary material. Electronic supplementary material, figure S1 shows the experimental timeline and summary of the key results.

After 14 days of acclimation, water samples were collected from the fish to establish their baseline cortisol level, using the method of water-borne cortisol extraction and quantification, as per Magierecka *et al*. [40]. Each fish was placed in a 600 ml beaker filled with 100 ml of water from the aquarium system for 30 min. According to previous research on sticklebacks [41], significant cortisol release into water does not occur until 60–90 min after exposure to a stressor and thus sampling for the first 30 min in this species provides reliable information on baseline cortisol levels. Collected water samples were processed through solid-phase extraction cartridges using vacuum manifold and the cortisol was extracted from the cartridges with 100% methanol. The extracts were evaporated under nitrogen gas, reconstituted in assay buffer and cortisol was quantified using a commercial colorimetric assay (ADI-900-071, Enzo Life Sciences, Exeter, UK).

Sticklebacks from the UCSP-exposed group were then exposed to the UCSP, which continued throughout the breeding season, i.e. until all females ceased to reproduce (67 days). The UCSP consisted of combinations of the following stressors: (i) lights turned on for 30 min during the dark period (night), (ii) lights turned off for 30 min during the light period (day), (iii) light intensity increased (480 lux to 1320 lux) for 30 min during the light period, (iv) lights turned off and bright light flashed in the darkness for 10 min during the light period, (v) tank aeration increased using an airstone to create water turbulence for 2 × 10 s, with a brief period of rest in between, (vi) shelter (artificial plant) removed for 15 min, (vii) fish chased with a net for 2 × 30 s, with 30 s of rest in between, (viii) fish captured and exposed to air in the net for 2 × 10 s, with 30 s of rest in between. The stressors were selected on the basis of being ethical, feasible to conduct in the aquarium setting and having been shown to induce stress effects in fish [42,43]. The UCSP schedule was created prior to the experiment by randomly selecting three of the above stressors for each day to ensure unpredictability. In addition, the timing of stressor presentation was also randomized, with one stressor applied at a time either in the morning (8 : 00–11 : 00), at noon (11 : 00–14 : 00) or in the afternoon (14 : 00–17 : 30). The exception was stressor 1, which was applied at night only. The shelves containing the UCSP tanks were fully shielded with opaque black plastic, and it was determined prior to the experiment that the Control fish would not be affected by the changes of the light regime. The UCSP induced changes in the feeding behaviour and activity of the exposed fish, with no indication of any habituation over the 67 days of exposure [40] and no difference in baseline cortisol level with respect to the duration of exposure (linear model (LM): *t*_1, 8_ = 0.229, *p* = 0.825; range of days under UCSP: 11–36).

### (b) F0 breeding and egg cortisol analysis

Prior to the start of the experiment, males in the non-stressed stock population that expressed nuptial coloration (red throat and blue eyes [44]) were placed individually in 10 l plastic tanks and provided with nesting material: sand and green polyester thread. This pool of mature males was subsequently used to fertilize clutches produced by the UCSP-exposed and Control females; only males that built a nest were used, and the pool was continually topped up with fresh mature males from the stock population. From the start of the UCSP, females in both the UCSP-exposed and Control groups were assessed visually each day for the signs of readiness to spawn (expanded abdomen and dilated anal papilla [44]); any such females were used for *in vitro* breeding immediately.

*In vitro* fertilization of clutches followed established protocols [45], with eggs stripped under light anaesthesia and half of each clutch fertilized with sperm from randomly selected stock males (see Appendix 2 of the electronic supplementary material for detailed protocol). Following egg stripping, females were released back into their home tanks, where they continued to be subjected to the original treatment (UCSP or Control), and the eggs from each clutch were placed in a separate tank. Egg stripping and fertilization were performed between 10 : 00 and 12 : 00 each day. The females were stripped when again gravid, until they produced a third clutch and/or until the end of the experiment (day 67 of the UCSP treatment). The remaining unfertilized eggs were used for cortisol quantification, performed on whole egg homogenates without prior extraction, using a commercial ELISA kit (see Appendix 3 of the electronic supplementary material for detailed protocol).

### (c) Clutch characteristics and fry size at hatching

The mean mass of a single egg was obtained by dividing total clutch mass by the number of eggs in a clutch. Mean egg volume was calculated using the formula volume = 4/3π*r*^3^, with radius (*r*) determined from photographs using ImageJ. Egg baskets were checked daily for hatching from day 8 post-fertilization. The date of hatching was recorded as the day on which the last fry hatched, with the checks performed at 10 : 00 daily. Temperature-corrected development time of each clutch, expressed as the number of accumulated thermal units (ATU), was determined from aquarium temperature records using the following formula: ATU = number of days between fertilization and hatching x average temperature (days to hatch range: 14–25; temperature range: 12.0–13.2°C). Upon hatching, all fry were removed from the tank into a Petri dish, counted and photographed on a lightbox with a piece of millimetre paper for scale. Mean fry size at hatching was calculated from measurements of standard length in 10 fry per clutch (or the maximum number surviving, whichever was the larger) using ImageJ processing software [46].

### (d) Fry measurement

The hatched F1 fry were retained in their original hatch tanks until the following spring. Initially they were held at 15°C and a photoperiod of 14L : 10D. This was reduced from mid-September 2017 by 0.5°C and 1 h per week, respectively, to 12°C and a photoperiod of 10L : 14D. On day 60 post-hatching (ph) the size of family groups was reduced where necessary to 15 fry due to space constraints. All F1 sticklebacks (regardless of parental treatment) were treated in the same way as the Control fish in the F0 generation, being subjected to standard husbandry practices but not to any additional stressors. See Appendix 4 of the electronic supplementary material for details on fry husbandry and reduction of the family size.

To determine the proportion of surviving fry and juveniles at various points of their development (relative to the survival at the previous time point), they were counted on days 14, 30, 60, 90 and 180 post-hatching (dph). At 30, 60 and 90 dph, fry were measured to determine their specific growth rate (SGR), calculated according to the following formula:

$$\mathrm{SGR}=100\times \left[\frac{\left(\ln(\mathrm{final}\;\mathrm{length})\text{–}\ln(\mathrm{initial}\;\mathrm{length})\right)}{\mathrm{no}.\;\mathrm{of}\;\mathrm{days}\;\mathrm{elapsed}}\right].$$

Fry were measured by transferring 10 individuals per clutch (or the maximum number surviving, whichever was the larger) into a dish filled with tank water and placed on a lightbox. The excess water was removed, so that all fry were at the same depth, and the fry photographed from above with millimetre paper for scale. The mean standard length of fry from each family group was determined using ImageJ. At day 180 ph, all individuals in the family group were lightly anaesthetized in benzocaine solution (25 mg l^−1^), weighed (to 0.001 g, after blotting dry) and measured (standard length to 0.1 mm).

### (e) F1 breeding

From mid-March 2018, the water temperature was increased by 0.5°C per week until it reached approximately 15°C and the photoperiod was changed to 14L : 10D (1 h per week) in order to bring fish into reproductive condition. To analyse whether chronic stress experienced by females from the F0 generation affected the reproductive strategy of their daughters (F1 generation), we used F1 families with at least three females that survived to sexual maturity. This resulted in the following sample sizes: 13 F1 full-sib families for the UCSP-exposed group came from an F0 female's first clutch, 12 from her second clutch and 10 from her third clutch (drawn from 13 F0 UCSP-exposed females in total); the corresponding sample sizes for the first, second and third clutches in Control group families were 15, 12 and 11 (from 16 F0 Control females).

From 1 April 2018, tanks were inspected visually on a daily basis to identify F1 females that were ready to spawn. The *in vitro* fertilization procedure, including selection of males for breeding, was the same as in the F0 population. To avoid any confounding effects of seasonality in F1 reproduction, only the first clutch of any F1 female was included in the study and females were separated from the family group following stripping. The inspection of tanks continued until up to three females per full-sib family had reproduced (i.e. up to nine F1 females derived from each F0 female), or until the end of the breeding season. Clutch size, mean mass of single eggs and mean size of F2 fry at hatching were determined as previously described.

### (f) Data analysis

Statistical analyses were performed in R (v.3.4.3, [47]), with linear mixed models (LMMs) fitted using the ‘lme4’ package [48] and *p*-values obtained using the ‘lmerTest’ package [49]. Statistical analyses for F0 were restricted to the females that produced at least two clutches, to allow for comparison between successive clutches produced by a female across the breeding season. Fish ID (or in the case of F2 models family ID, being a combination of F0 fish ID and clutch number) was included in the models as a random factor due to multiple measurements from the same female. Non-significant terms were removed by backwards selection. The fixed factors included in all initial models are specified in the electronic supplementary material, table S1. Electronic supplementary material, tables S2–S5 provide detailed information on sample sizes for each combination of treatment and clutch number.

A LMM was used to analyse differences in egg cortisol levels between the two F0 treatment groups (final model structure: egg cortisol∼ treatment + clutch + (1|ID); UCSP-exposed *N* = 16, Control *N* = 32). Clutch size in the two treatment groups was analysed using a generalized linear mixed model (GLMM) with Poisson distribution (clutch size ∼ clutch + (1|ID); UCSP-exposed *N* = 52, Control *N* = 54). Initially, egg cortisol concentration was included as a fixed factor in the clutch size model, but this was shown to be non-significant; since measurements of egg cortisol were not available for all clutches the variable was not included in further analyses in order to maximize the sample size. Similarly, egg cortisol was not included in the LMM of single egg mass (egg mass ∼ treatment + clutch + treatment:clutchsize + (1|ID); UCSP-exposed *N* = 51, Control *N* = 53). Single egg mass values were log-transformed to achieve a normal distribution of the residuals.

A LM was used to analyse the size of fry at hatching, restricting the sample size to females for which the information on fry size was available from at least two clutches (fry size ∼ treatment + clutch + female mass + egg volume + treatment : female mass + clutch : egg volume; UCSP-exposed *N* = 38, Control *N* = 43). Initially, Tank ID and Fish ID were included as random factors and an LMM model was used. However, the variance of the random effects was estimated as 0 (resulting in a singular model fit) and they were thus dropped without affecting model estimates.

Five separate GLMMs with a binomial distribution were used to analyse differences in the survival of F1 fry produced by mothers from the two treatment groups, relative to the survival at the previous time point . Sample size variation was due to missing data at different time points (UCSP-exposed *N* = 38–39, Control *N* = 42–44), with the exact sample sizes specified in table S3 of the electronic supplementary material. The response variable in these models was the proportion of fry in a family that were alive at each time point. To control for the relative influence of the number of fry in a family on the response variable, we used a ‘weights’ argument in the model fitting process, where ‘weights’ is the number of fry in the family (surviving + dead) used to generate each proportion. A LM was used to analyse differences in the growth rate (SGR) of F1 fry produced by F0 mothers from the two treatment groups (SGR ∼ clutch + timepoint + initial length + group size + clutch : timepoint; UCSP-exposed *N* = 40, Control *N* = 45) at different time points (1–30 dph, 30–60 dph, 60–90 dph and 90–180 dph).

A GLMM with Poisson distribution was used to analyse the sizes of F2 clutches (clutch size ∼ F0 treatment + female clutch of origin + female mass + Julian date of fertilization + F0 treatment : female mass + (1|Family ID); UCSP-exposed *N* = 84, Control *N* = 80); these clutches were produced by F1 mothers originating from the three successive breeding attempts of F0 females from the two treatment groups. A LMM was also used for analysis of single egg mass (egg mass ∼ F0 treatment x female clutch of origin + Julian date of fertilization + F0 treatment x female mass + (1|Family ID); UCSP-exposed *N* = 84, Control *N* = 88) and the size at hatching (fry size ∼ F0 treatment + female clutch of origin + development time + F0 treatment : female clutch + F0 treatment : development time + (1|Family ID); UCSP-exposed *N* = 82, Control *N* = 71) of F2 fry.

## 3. Results

### (a) Traits of eggs produced by F0 females

Preliminary analysis of egg cortisol concentration revealed the presence of one clutch with abnormal residuals (greater than 3 s.d. from the mean), which affected the distribution of model residuals. This outlier was removed, and data re-analysed, with the results of the refined analysis being qualitatively similar to the original analysis that included the full dataset. UCSP-exposed females did not deposit more cortisol in their eggs than Controls (LMM: *F*_1, 18_ = 0.314, *p* = 0.582, electronic supplementary material, figure S2). Neither female mass (*F*_1,17_ = 0.648, *p* = 0.432) nor female baseline cortisol level (*F*_1, 17_ = 1.161, *p* = 0.296) had an effect on the level of cortisol in eggs.

**Figure 1.**
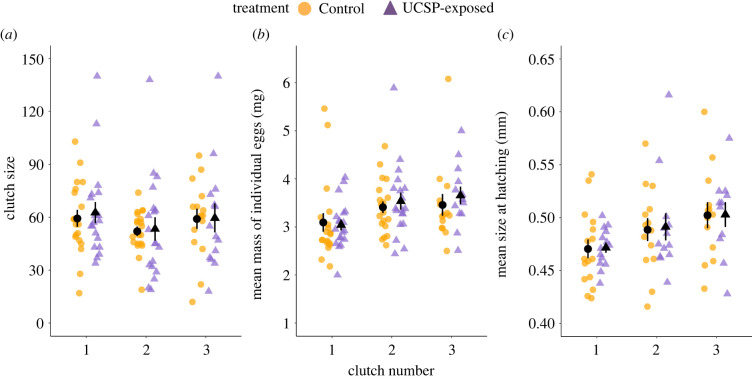
Variation in (*a*) clutch size, (*b*) mean mass of an individual egg (mg) and (*c*) mean fry size at hatching (mm) in three successive clutches produced across the breeding season by female three-spined sticklebacks from Control (circles) and UCSP-exposed (triangles) treatment groups. The figures show individual data points for each clutch, with means ± s.e.; sample sizes are provided in the electronic supplementary material, table S2. (Online version in colour.)

The average clutch size of UCSP-exposed females did not differ from that of Controls (Wald chi-squared test: *χ*^2^ (1, *N* = 106) = 0.02, *p* = 0.885, figure 1*a*), nor did the egg mass (LMM: *F*_1, 63_ = 2.729, *p* = 0.103); the parameter estimates for both models are given in the electronic supplementary material, tables S6 and S7. However, there was a significant increase in the mass of an egg throughout the breeding season, with eggs from later clutches being heavier (*F*_2, 67_ = 7.179, *p* = 0.001, figure 1*b*). The mean size at hatching of offspring from UCSP-exposed females did not differ significantly from that of offspring from Controls (LM: *F*_1, 72_ = 0.035, *p* = 0.853), but fry hatching from eggs laid later in the season were significantly larger than those from early and middle clutches (*F*_2, 72_ = 6.226, *p* = 0.003, figure 1*c*; electronic supplementary material, table S8).

### (b) F1 survival and growth rates

**Figure 2.**
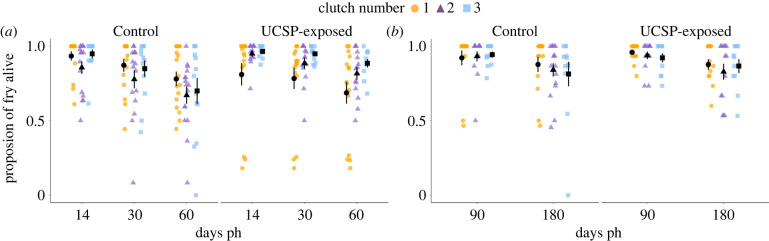
Three-spined stickleback F1 fry survival (as proportion of initial fry alive at each time point, relative to the previous time point) in three successive clutches produced across the breeding season by F0 females from Control and UCSP-exposed treatment groups; (*a*) shows survival between 0 and 14 days post-hatching (dph), between 14 and 30 dph and between 30 and 60 dph and (*b*) shows survival between 90 and 180 dph. The figures show individual data points for each clutch with means ± s.e.; sample sizes are provided in the electronic supplementary material, table S3. Survival at 90 and 180 dph follows the reduction of the family size to ≤15 fry at 60 dph (see text for details). (Online version in colour.)

Exposure of a female to a period of chronic stress altered the survival trajectories of her offspring from successive clutches produced across the breeding season. At 14 dph, there was an overall significant interaction between F0 treatment and clutch number (Wald chi-squared test: *χ*^2^ (2, *N* = 86) = 56.87, *p* < 0.001; figure 2; electronic supplementary material, table S9). The difference was apparent with regard to Clutch 2, which in the UCSP-exposed group had higher survival than Clutch 1 (and similar to that of Clutch 3), whereas in the Control group Clutch 2 had the lowest survival of the three clutches. The interaction between F0 treatment and clutch number was also significant at 30 dph (*χ*^2^ (2, *N* = 86) = 52.92, *p* < 0.001) and 60 dph (*χ*^2^ (2, *N* = 86) = 34.93, *p* < 0.001): while in the UCSP-exposed group, fry from a female's first clutch had lower survival than those from later clutches, in the Control group, these first-produced fry had the highest survival. The direction of this effect changed at 90 dph, with clutches produced later in the breeding season (Clutch 3) having significantly lower offspring survival in the UCSP-exposed group, but significantly higher offspring survival in the Control group (interaction between F0 treatment and clutch number, Wald chi-squared test: *χ*^2^ (2, *N* = 84) = 6.07, *p* = 0.048). Any differences between treatment groups and clutches were no longer significant by day 180 ph. Please refer to electronic supplementary material, table S9 for parameter estimates of inter-clutch comparisons.

**Figure 3.**
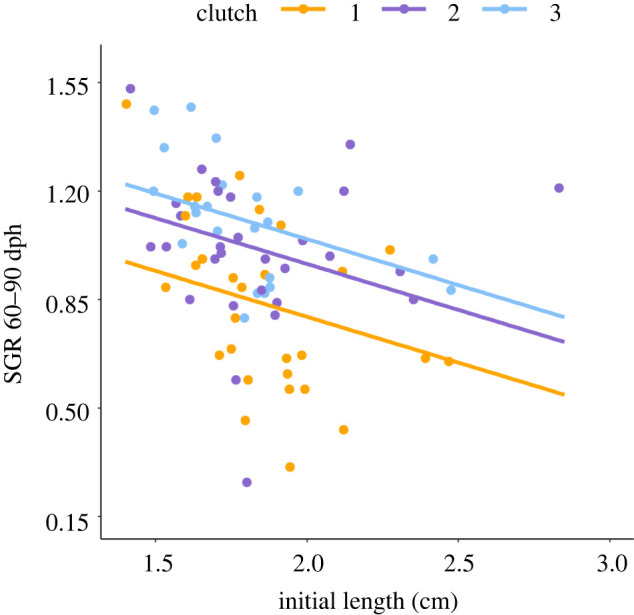
SGR (% length increase per day) of three-spined stickleback fry between 60 and 90 days post-hatching (dph). SGR is plotted against initial length (cm) at the beginning of the time period. Data are shown for fry from three successive clutches produced by females across the breeding season (combined for the Control and UCSP-exposed fish). The sample size is provided in the electronic supplementary material, table S4. The regression line was predicted from a model including treatment group, clutch number and initial length as fixed effects; see text and electronic supplementary material, table S10 for details. (Online version in colour.)

The SGR of F1 fish did not differ between the offspring of the UCSP-exposed and Control F0 females (LM: *F*_1, 310_ = 0.172, *p* = 0.678; electronic supplementary material, table S10), but there was a significant interaction between the period of measurement and clutch of origin (LM: *F*_6, 310_ = 3.940, *p* < 0.001). This was due to fry from Clutch 2 and 3 growing at a faster rate between 60 and 90 dph than fry from Clutch 1 (Clutch 2: *t*_6, 310_ = 2.877, *p* = 0.004, Clutch 3: *t*_6, 310_ = 4.340, *p* < 0.001), regardless of the maternal treatment (figure 3; electronic supplementary material, table S10).

### (c) Traits of eggs produced by F1 females

**Figure 4.**
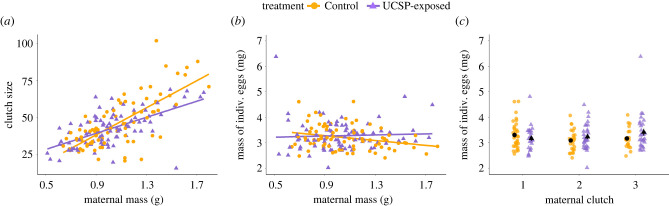
The relationship between mass (g) of F1 female three-spined sticklebacks and their (*a*) clutch size and (*b*) egg size (mg). (*c*) The mean mass of an individual F2 egg of clutches produced by F1 females originating from the first, the second or the third clutch of F0 females. The F1 females were not subjected to stressors, but were the offspring of either UCSP-exposed or Control F0 mothers. The regression lines in (*a*) and (*b*) were fitted using linear smoothing; see text and electronic supplementary material, table S11 for details. The figures show individual data points; (*c*) also shows means ± s.e. for each clutch (please note that the error surrounding the means is small and may thus not be evident in the figure: Control group, Clutch 1 s.e. = 0.093, Clutch 2 s.e. = 0.082, Clutch 3 s.e. = 0.087; UCSP-exposed group Clutch 1 s.e. = 0.098, Clutch 2 s.e. = 0.093, Clutch 3 s.e. = 0.133). Sample sizes are provided in the electronic supplementary material, table S5. (Online version in colour.)

The average clutch size produced by F1 daughters of UCSP-exposed mothers did not differ from that of Control daughters (Wald chi-squared test: *χ*^2^ (1, *N* = 164) = 1.09, *p* = 0.296). However, there was a significant interaction between F0 treatment group and the somatic mass of the F1 female (Wald chi-squared test: *χ*^2^ (1, *N* = 164) = 9.61, *p* = 0.002), with heavy F1 females (greater than 1.1 g) from the UCSP-exposed group producing smaller clutches relative to Control females of the same body mass; figure 4*a*; electronic supplementary material, table S11). F1 females produced late in the season (Clutch 3) by UCSP-exposed mothers had significantly heavier eggs than the F1 daughters of Control females from the corresponding clutch (LMM for treatment x clutch interaction: *F*_2, 52_ = 2.286, *p* = 0.112; please note that this was not significant for Clutch 2, hence the lack of significance of the overall interaction: see electronic supplementary material, table S11 for details; figure 4*c*). Moreover, exposure of F0 females to a period of chronic stress influenced the relationship between the mass of an F1 female and the individual mass of the F2 eggs that she produced (LMM: *F*_1, 132_ = 8.444, *p* = 0.004): heavy F1 females (greater than 1.1 g) from the UCSP-exposed group produced heavier eggs than did F1 females of the same body mass originating from the Control group (figure 4*b*; electronic supplementary material, table S11).

**Figure 5.**
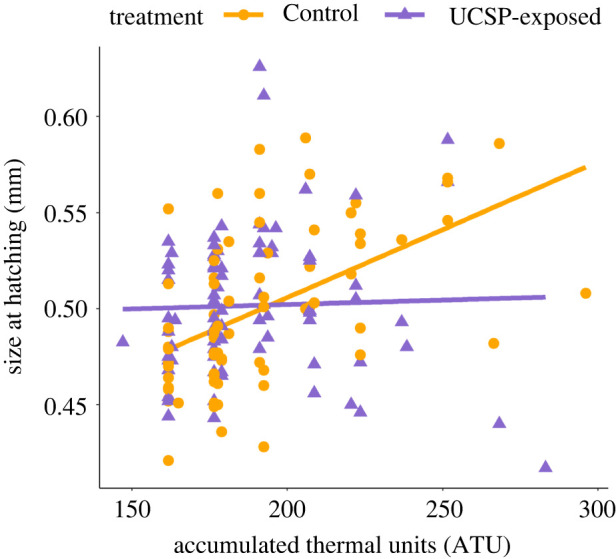
The relationship between mean size at hatching (in mm) and development time (as ATU) of F2 three-spined stickleback fry. Their mothers (F1) were not subjected to stressors, but were the offspring of either UCSP-exposed or Control F0 females. The sample size is provided in electronic supplementary material, table S5. The regression lines were fitted using linear smoothing. Note that higher ATU values indicate slower development. (Online version in colour.)

A significant interaction between F0 treatment and F1 clutch of origin (LMM: *F*_2, 55_ = 4.920, *p* = 0.011; electronic supplementary material, table S12) indicated that F1 females that were spawned later in the breeding season by UCSP-exposed F0 mothers produced F2 fry that were larger at hatching than corresponding fry from the Control group. The size of F2 fry at hatching was also significantly influenced by the interaction between F0 treatment and F2 development time (*F*_1, 143_ = 11.532, *p* < 0.001; electronic supplementary material, table S12). The size of F2 fry originating from Control F0 females increased with time spent developing, i.e. the fry with higher ATU value were larger at hatching. However, the F2 fry originating from the UCSP-exposed F0 females showed no relationship between these two factors, with size at hatching being constant, regardless of the development time (figure 5).

## 4. Discussion

In this paper, we demonstrate that in female sticklebacks the seasonal change in the trade-off between reproduction and self-maintenance was a stronger driving force in shaping of reproductive strategy than unpredictability of the environment. Thus, seasonality may override the effects of chronic exposure to stressors, as seen in the analysis of the egg mass and the size of fry at hatching. Similarly, seasonality rather than maternal experience influenced the rate of early offspring growth. We also provide a novel insight into the combined transgenerational effects of chronic stress and seasonality of reproduction on offspring survival and reproductive strategy; key results are summarized in the electronic supplementary material, figure S1.

Despite the lack of effect of maternal stress exposure on clutch size and fry size at hatching, there was variation over the course of the breeding season in reproductive allocation by females in both treatments. This was manifested in considerable inter-clutch variation in the number of eggs produced and size of fry at hatching. Fish using an annual mode of reproduction show shifts in the trade-off between reproduction and self-maintenance, increasing their reproductive investment towards the end of the breeding season [50]. It is thus expected that the females attempted to maximize their fitness by allocating more resources in their later eggs (reflected in the mass of individual eggs and size of the resulting offspring at hatching) as an example of an anticipatory maternal effect. Overwinter survival of juveniles is related to their body size and condition, and so as the time available for them to grow prior to winter diminishes, investing in larger eggs and larger offspring will increase their chance of survival [51]. In addition, the earlier clutches were produced by females that have just reached sexual maturity but possibly did not achieve their peak reproductive capacity. Thus, the seasonal increase in reproductive allocation may result from the combination of a trade-off between reproduction and self-maintenance and improvement of the reproductive capacity over the female's reproductive lifespan.

We found no effect of maternal stress on offspring growth rate, but there was evidence of an inter-clutch difference in growth pattern, with late-produced offspring growing faster between two and three months post-hatching. Along with our observation of an increase in egg size in clutches produced later in the breeding season, this is consistent with the hypothesis of an increased maternal allocation to allow late-produced offspring to reach larger size before the onset of winter. A potential confounding factor here would be the possibility that maternal allocation later in the breeding season was linked to longer exposure to the UCSP and not just to seasonality. Since there was no effect of the duration of exposure on baseline cortisol level and no behavioural habituation to the UCSP [40], it is likely that the observed effects are due to seasonality; however, this requires further testing, e.g. by staggering the start of the exposure across the breeding season.

Growth rate is a further example of a trait found to be more affected by seasonal maternal effects than any effect of maternal stress. Nonetheless, seasonal and stress-induced maternal effects are not always mutually exclusive. Seasonal maternal adjustments to increase the probability of survival of later clutches have previously been documented [52]. In addition, maternal allocation strategy may be influenced by environmental conditions. If these conditions are stable, females can reliably anticipate the environment that their offspring will encounter upon hatching/birth and adjust their phenotype accordingly [53]. In the present study, the quality of offspring (indicated by their survival rate in the first three months) of Control females declined across the breeding season, while females exposed to chronic stressors produced higher-quality offspring later in the season. However, later in life the offspring of UCSP-exposed females had lower survival than those of Controls. Where long-term exposure to stressors provides females with reliable information on the anticipated offspring environment, adjustment of the offspring phenotype can provide a survival advantage [53]. Here the offspring did not experience the same unpredictable environment as their mothers, thus the resulting phenotype may not have been the most advantageous in the long term, leading to an increased mortality after the first three months. However, it must be noted that this study provides an insight into the effects of chronic stress on wild fish in a laboratory setting, using stressors relevant in this context. To fully understand the adaptive potential of maternal stress on offspring phenotype across contexts, future studies may need to shift the focus to chronic stressors that are more ecologically relevant in a natural setting.

In terms of transgenerational effects of chronic exposure to stressors in F0 females, we found a complex relationship between a female's exposure to chronic stress, the body mass of her daughters at sexual maturity and the number and mass of eggs these daughters produced. Heavier daughters of UCSP-exposed fish were relatively less productive than the daughters of Control F0 fish, which is at odds with the theory predicting that additional resources (which heavier females potentially possess) should be invested in increasing individual productivity [54,55]. Both UCSP-exposed and Control females from the maternal population (F0) produced heavier eggs later in the breeding season, which is consistent with the theory of increased investment late in the reproductive lifespan [50]. However, F1 females originating from the clutches produced late in the breeding season produced heavier eggs, but only if their mothers were exposed to chronically stressful conditions. Individuals that receive a greater pre-natal investment are able to invest more resources into their own gametes [56,57]; it is however unclear why this effect was only apparent in the offspring of the stress-exposed sticklebacks. We propose that in this study the observed effect may result from interplay between increased maternal provisioning at the end of the breeding season and an anticipatory intergenerational effect. However, since late clutches were produced by mothers who were exposed to the UCSP for longer, this hypothesis requires further rigorous testing to disentangle the effects of seasonality from a potential confounding effect of the duration of exposure. Moreover, the relationship between egg quantity and size in F1 females originating from UCSP-exposed and Control treatments may result from different strategies adopted by these females, i.e. few large (UCSP-exposed) versus many small (Control) eggs. Assuming that being large is beneficial in an unpredictable environment, it would be an adaptive maternal strategy to produce larger offspring upon exposure to the UCSP. This is, however, at odds with the results of Shama [58], who reported smaller egg size in sticklebacks exposed to unpredictable environmental conditions.

In addition to the effect on clutch and egg size, we also found that grandmaternal treatment affected the size of F2 fry at hatching. In the F0 generation, an increase in size at hatching was observed with each successive clutch produced by females from both treatment groups, suggesting maximization of maternal allocation towards the end of the breeding season. F1 females originating from late-produced clutches gave rise to F2 offspring that were larger at hatching, but only if their grandmother (i.e. the F0 female) was subjected to chronically stressful environmental conditions. In vertebrates, larger size at hatching/birth is generally positively correlated with survival and may provide a survival advantage in stressful conditions ([59,60] but see [58]). Therefore, the observed greater size at hatching of sticklebacks whose grandmothers experienced stressful conditions may be an example of an anticipatory effect persisting across generations. An additional argument in favour of this hypothesis can be found in the relationship between developmental rate and fry size at hatching, with newly hatched F2 sticklebacks originating from stress-exposed grandmothers being relatively large, even if their development time was relatively short. Accelerated early growth and development can lead to an array of negative effects, including increases in oxidative damage and shortening of telomeres [61,62]. These in turn can lead to reduced probability of survival [63], accelerated ageing [64] and reduced lifespan [65]. Therefore, an increased rate of pre-natal growth is only beneficial if the offspring are of high quality and can withstand or offset the negative effects of accelerated growth, or if there are disproportionate advantages to having a large size at hatching.

## 5. Conclusion

What emerges from these results is that the relationships between various maternal and egg/offspring characteristics are complex and that their complexity may increase across generations, for example due to added confounding factors resulting from non-matching maternal and offspring environments. Moreover, some effects of exposure to chronically stressful environments may be unclear in the first generation but may become more explicit in later generations; these processes can interact with seasonal patterns in reproductive investment to have a different effect on offspring from successive breeding attempts. Therefore, when assessing the implications of chronic environmental stressors, it may be important to look not only at multiple generations, but also at multiple cohorts produced within a generation, as examining one reproductive attempt appears to only to provide a snapshot of the processes resulting from maternal exposure to stressors.

## Data Availability

The data and code associated with this publication are available from Figshare (https://doi.org/10.6084/m9.figshare.17099243) [66]. Electronic supplementary material is available online from Figshare (https://doi.org/10.6084/m9.figshare.17099300) [67].
